# ‘Sometimes I’m feeling baffled and they’re probably feeling baffled’: On the experiences of psychological therapists working with autistic people in a structured primary care service for anxiety disorders and depression

**DOI:** 10.1177/13623613251341610

**Published:** 2025-05-22

**Authors:** Paul K Miller, Samantha LJ Bowden, Natalie Dewison, Barry Ingham, Richard Thwaites, Dave Dagnan

**Affiliations:** 1 University of Cumbria, UK; 2 Lancashire and South Cumbria NHS Foundation Trust, UK; 3 University of Glasgow, UK; 4 Cumbria, Northumberland, Tyne and Wear NHS Foundation Trust, UK

**Keywords:** adults, anxiety, autism spectrum disorders, communication and language, depression, interventions – psychosocial/behavioural, qualitative research

## Abstract

Autistic people are more likely to experience mental health problems such as anxiety disorder and depression than are the general population. This study reports a qualitative analysis of interview data provided by 12 psychological therapists regarding their experiences of working with autistic people with anxiety disorders and/or depression within a structured primary care mental health service in the north of England. Interviews were analysed using thematic analysis. Four main themes were identified: (1) Experience and Trepidation, (2) Wrong Service, Only Service, (3) Therapeutic Environment and (4) Training and Adaptations. Participating therapists identified challenges in the structure of the services they worked in, the applicability of conventional therapies and the need for autism-specific therapy training ideally led by autistic people. Centrally, participants did not routinely feel fully equipped to separate endemic aspects of autism itself from features of a mental health disorder in an autistic person, which had left some feeling powerless to help in certain cases, or as if they may have done more harm than good. All participants were, however, able to identify positive adaptations made from practical experience, and most reported a growing confidence in working with autistic people.

**Lay Abstract:**

**The experiences of psychological therapists working with autistic people in a primary care service for anxiety disorders and depression**

We are a group of autistic people, academic researchers and psychological therapists, with some of us being more than one of those things. We started from the knowledge that autistic people are particularly prone to have anxiety disorders and depression. We were, therefore, interested in how current ‘talking therapy’ services in England might, or might not, be helping autistic people with those problems. To address this issue, we interviewed 12 psychological therapists in the north of England who had experience of working with autistic people with an anxiety disorder, depression or both. We found that the therapists often felt that they were not prepared or trained to give autistic people their best service. The therapists were also concerned that some of the therapies they usually applied did not always work with autistic people, or sometimes even made things worse. They felt it was important, however, that autistic people should keep using the service, as there was no other service available to them if they had an anxiety disorder or depression. There was evidence, however, that talking therapies still had positive effects for autistic people, and that therapists had therefore probably underestimated their positive impact in a lot of cases. Consequently, training was recommended such that psychological therapists might better understand mental health and specific therapy adaptations that help autistic people.

## Introduction

Autistic people are more likely to experience anxiety disorders and depression than the general population (O’Nions et al., 2024). For example, using primary care data from Wales, UK, Underwood et al. (2023) report clinically diagnosed anxiety disorders as present in 27.56% of autistic people compared to 13.60% of a matched sample of people who were not autistic, and depression at rates of 31.39% compared to 17.90%. Autistic people also have a greater likelihood of needing mental health services than people who are not autistic, but they report greater difficulties in accessing these services and lower satisfaction with them (Adams & Young, 2021; Hirvikoski et al., 2016; Mason et al., 2019).

Recent reviews have reported inconclusive results for the efficacy of psychological therapies such as cognitive behaviour therapy (CBT) and behavioural activation (BA) when applied to autistic adults with anxiety disorders and/or depression (Binnie & Blainey, 2013; Menezes et al., 2022; Weston et al., 2016; Wichers et al., 2023). However, specific protocols have been developed for autistic people experiencing anxiety disorders (Rodgers et al., 2023, 2024) and depression (Russell et al., 2020), and CBT-based psychological therapies remain the recommended interventions in the United Kingdom for autistic and non-autistic populations alike (National Institute for Health and Care Excellence [NICE], 2021). In England, where training regarding work with autistic people and/or people with an intellectual disability is now a mandatory consideration for all public-sector Health and Social Care workers (National Development Team for Inclusion, 2022), recent research has indicated that talking therapies can be effective for autistic people with anxiety disorders and/or depression, but not necessarily as effective as they are for others. As a case in point, El Baou et al. (2023) reported therapeutic outcomes for 8593 autistic people in England. While outcomes were positive (56.1% improvement greater than the reliable change index of the measures used), they were less positive than those for a matched sample of people who were not autistic (61.7%).

Extant research has also addressed how therapy and services can be adapted to be more suitable for autistic people and how therapists can improve their practice when working with autistic people (Cooper et al., 2018; Lipinski et al., 2022; Spain et al., 2023). For example, Riches et al. (2023) interviewed clinical psychologists working within a specialist autism service and highlight the perceived importance of key interpersonal adaptations (e.g. understanding the client’s communication style, using visual prompts and working on emotional literacy) to support development of the therapeutic relationship. Recently developed consensus guidelines recommend similar adjustments and adaptations (National Autistic Society, 2021; Rodgers et al., 2023; Russell et al., 2020).

In England, psychological therapy for anxiety disorders and/or depression is primarily provided through NHS Talking Therapies for Anxiety and Depression (NHS TTad; previously known as Improving Access to Psychological Therapies, or IAPT). TTad services are available in all parts of England, and in 2022–2023 received 1.76 million referrals, of which 672,193 completed treatment (NHS England, 2024). The *NHS Talking Therapies for Anxiety and Depression Manual v7* (National Collaborating Centre for Mental Health, 2024) states that these services are guided by three central principles:

1. The provision of NICE recommended evidence-based psychological therapies in a stepped care model, through:
2. An appropriately trained and accredited workforce, who receive weekly outcome focussed supervision from senior clinicians with the correct competencies to support them, and
3. Outcome measurement on a session-by-session basis, which both structure the person’s treatment and supports service improvement.

In the stepped care model, people with mild to moderate depression, and/or anxiety disorders, are typically offered low-intensity treatment from registered psychological well-being practitioners (PWPs). People who do not recover sufficiently can be ‘stepped up’ to high-intensity treatment delivered by registered high-intensity therapists (HITs). NHS TTad services have several additional priorities, including to reduce inequalities in groups that are known to have poorer access to conventional services, and/or outcomes from them (National Collaborating Centre for Mental Health, 2024). The NHS TTad model is structured for high-volume treatment of the general population, and it is likely that adaptations would be needed to meet the needs of autistic people.

The aim of this study is to explore the experiences of psychological therapists working with autistic people within NHS TTad. It is hoped that the study will inform the development of structured therapy services and increase their accessibility and effectiveness for autistic people. Moreover, the English NHS TTad model has formed the basis for services elsewhere, including Australia (Baigent et al., 2023), Canada (Vincent et al., 2021), New Zealand (Haarhoff & Williams, 2017), Spain (Cano-Vindel et al., 2022) and Norway (Knapstad et al., 2018). The experiences of therapists working in the English system are, therefore, likely to be of interest beyond those working in the English system itself.

## Method

This is a qualitative study based on semi-structured interviews with registered psychological therapists, all of whom were working in NHS TTad services across a geographically large and demographically diverse area in the north of England, encompassing both urban and rural contexts.

### Recruitment

The criteria for participants to be included in the study were as follows:

1. Psychological practitioners with registration and/or accreditation relevant to their current role in NHS TTad services (including CBT therapists, psychological well-being practitioners, counsellors and so on; see National Collaborating Centre for Mental Health, 2024).
2. A minimum of 2 years of post-qualification experience of working in NHS TTad services.
3. Self-reported experience of working directly with at least one autistic adult as part of this service.

An email invitation detailing the purposes and requirements of the project and criteria for inclusion was cascaded to potential participants in seven NHS TTad services via service managers. The first 12 therapists to return informed consent were recruited.

### Participants

Table 1 describes the key demographic characteristics of the participants, eight of whom identified as female and four as male, with an age range of 37–63 years and an experience range of 2–15 years working in NHS TTad services. All participants reported specialising in one or two of the following therapeutic modalities: Cognitive Behavioural Therapy (CBT), Eye Movement Desensitisation and Reprocessing (EMDR), Interpersonal Therapy, Low Intensity CBT and/or Person-Centred Experiential Counselling.

**Table 1.** Demographic information for study participants.

Participant	Gender	Age (years)	Experience (years)	Specialist therapeutic modality/modalities	Interview length (min)
1	Female	57	10	Person-Centred Experiential Counselling	45
2	Male	39	2	Interpersonal Therapy	35
3	Female	40	9	Cognitive Behavioural Therapy, Interpersonal Therapy	58
4	Male	37	4	Person-Centred Experiential Counselling, Interpersonal Therapy	47
5	Female	52	5	Cognitive Behavioural Therapy	49
6	Male	61	7	Cognitive Behavioural Therapy, Eye Movement Desensitisation and Reprocessing	57
7	Female	44	4	Low Intensity Cognitive Behavioural Therapy	47
8	Female	62	7	Cognitive Behavioural Therapy	38
9	Male	56	6	Cognitive Behavioural Therapy	42
10	Female	63	12	Cognitive Behavioural Therapy, Eye Movement Desensitisation and Reprocessing	47
11	Female	39	5	Low Intensity Cognitive Behavioural Therapy	45
12	Female	57	15	Cognitive Behavioural Therapy, Eye Movement Desensitisation and Reprocessing	45

Participants were not directly asked to estimate the number of autistic people with whom they had worked, as senses of how ‘experienced’ they felt around this matter (and why) were key features of the interviews themselves. Participants were also not asked to declare in advance whether they were themselves autistic, although ample opportunity was afforded during the interviews to do so if they wished. None made relevant a diagnosis at any point.

### Procedure

The interviews (duration range = 35–58 min; mean duration = 46 min) followed a semi-structured topic guide, available as Supplementary Material to this article, with agreed prompts where useful in developing topic-salient narratives. All interviews were conducted and recorded online by the second author, via Microsoft Teams, and subsequently transcribed by the same author. The transcribed data amounted to approximately 83,000 words. Redactions for identity protection were made at the point of transcription (primarily names, places and exact times/dates).

### Data analysis

Analysis of data used Braun and Clarke’s (2022) thematic approach. Initial data familiarisation was undertaken by the first and second authors, with both making observational notes to help direct prospective coding. The second author then conducted provisional coding upon all transcripts, which was reviewed and refined with the first author over a series of rounds. A set of interim themes, and a thematic map, was then worked up by the first author and verified for correspondence with the original data by the second author. Thereafter, the first and second authors further refined the analysis for consistency with the source materials, selecting key extracts for illustration, until full consensus on the analytic product (including appropriate theme names) was reached. The broader research team subsequently reviewed the analysis for internal consistency, and for veracity, and this input was incorporated into a further revision of the analysis. Key changes at this analytic stage related chiefly to (a) naming and describing the higher-order themes, such that they would speak more organically to therapists themselves, and (b) selecting extracts and including additional details that more clearly articulated the character of given therapies for a non-expert audience.

### Community involvement statement

Three of the authors of this article are working psychological therapists, two are autistic and all are experienced researchers. These positions, in collaboration, shaped the design and execution of the research at all levels. The interview schedule was also reviewed by an autistic advocate with experience of NHS TTad services, who did not propose any necessary changes.

## Results

Thematic analysis of the interviews with NHS TTad therapists yielded four main themes:

1. Experience and Trepidation;
2. Wrong Service, Only Service;
3. Therapeutic Environment;
4. Training and Adaptations.

**Figure 1.**
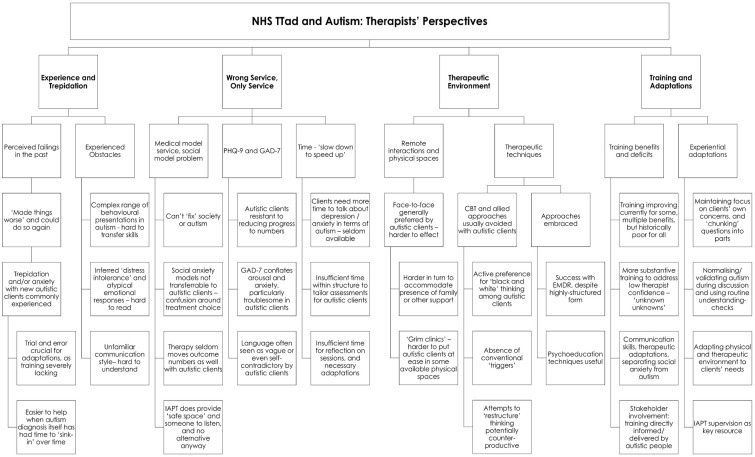
Thematic analysis of qualitative data.

The relationship between these and the branching subthemes from which they were inductively assembled is schematised in Figure 1.

This analysis is outlined in greater depth below, with reference to data where illustrative.

### Theme 1: experience and trepidation

All participants described a concern with ‘past failings’, which was largely attributed to a lack of autism-specific training leaving distinct gaps in their knowledge and therapeutic skill. Participants identified cases where they had either not helped or even worsened a client’s situation. Some therapists gave examples where undiagnosed (or undisclosed) autism had not been recognised at all:

PT1: I can now think back to certain clients in the past that I would now be querying would be autistic . . . They would have neurodiversity that I did not pick up at the time . . . I think that’s a failure of my knowledge and within the system . . . it’s failed those people . . . I think needed that support.

Other therapists described clients known to be autistic, but where the participant struggled to help them with their specific mental health condition(s):

PT8: [There] was one that I think I particularly failed . . . he had . . . he had been diagnosed with autism. He was extremely serious in this presentation . . . He’d come with all his notes. He was very, very sort of organised, lovely guy, but . . . I just never felt he got off the ground. I didn’t know what I was doing. I didn’t feel as though I made any change.

However, a well-established autism diagnosis was seen as more helpful when implementing adaptations with autistic people:

P10: [T]he person I’m working with at the moment, who’s had the diagnosis for a few years, I’m finding it easier to work with him . . . he’s aware of it and kind of has acknowledged it and understands it quite well. And so that’s allowed us to kind of manage you know, his autism in the sessions quite proactively.

Participants articulated three core concerns when working with autistic people. The first was to acknowledge autistic people as individuals with commonalities and differences compared to other autistic people, meaning that the adaptations required may vary:

PT1: [O]ne person with autism is probably different to every other person. They’ll be similar, they’ll be similarities within it. But then, a lot of difference from the same and the same level as well.

The second concern was that autistic people’s emotional responses may be different to those of people who are not autistic:

PT5: [S]o, on the one hand, [the client] is saying, I don’t think it’s a problem. I’ve dealt with this, and then she’s very distressed. So, I was very confused thinking ‘Okay clearly there is . . . something here’. . . . I constantly felt wrong-footed with her emotional responses which were different to the words she was saying.

Finally, and closely linked to emotional expression, some participants found that the communication and interaction differences of some autistic people created early barriers within therapy:

PT6: [I’m] thinking ‘I’m really struggling to understand what you’re telling me’. Like they’re literally speaking a different language to me . . . Sometimes I’m feeling baffled and they’re probably feeling baffled and confused, and it just feels like there’s a pane of glass between us and . . . we’re really, really struggling and that’s quite frustrating for both of us.

### Theme 2: wrong service, only service

Many therapists reported that the structural organisation of NHS TTad services was unhelpful for some autistic people. The most fundamental concern was that NHS TTad involves time-limited and structured interventions and focussed on anxiety disorder or depression. This was viewed as problematic when working with autistic people, as several participants noted that autistic people hope to gain understanding of their autism or to get support to make psychosocial changes to the person’s world:

PT3: [It can] be then quite difficult because then [the client enters] this service thinking [they] might get some support for these issues, but it’s not a mental health issue and therefore we don’t work with that.

Under these circumstances, many established therapeutic assumptions for treatment of depression and anxiety disorder can break down:

PT8: The assumption that this [autistic] person needs to be out in the world doing things with other people, that assumption from me was unhelpful for them, I think.

Nowhere were these issues more keenly articulated than with respect to social anxiety, which was widely seen as particularly difficult to ‘separate’ from autism:

PT3: [She] will sit quite small, and sometimes that is part of social anxiety and sometimes that’s related to, I guess, the autism. [What] she talks about is feeling often overstimulated and worried about what people think of her in a social situation, it’s really difficult as a therapist to pull apart to a certain extent, to know which bit we’re treating and also to know from a kind of conceptualization type whether the patient would even know the difference and whether that [even] matters.

Although there were concerns about the service model, therapists generally concluded that NHS TTad was providing a valuable service, even where it was not always effective through its intended mechanisms:

PT10: That was an important part of the sessions for her, just talking about how it felt to be her. Probably more of that than CBT, to be honest in those sessions.

Perhaps more importantly, NHS TTad was seen as valuable because there were few (if any) available alternative services for autistic people:

PT6: I do think some of this work is sounding . . . more long term, with [TTAD] being required to somehow take the strain because there’s no other service taking the strain for supporting people with autism.

There were also key reservations about how well the measures used in sessional assessment worked for autistic people. The first concern was a reported challenge in using the scaling in anxiety disorders and depression measures:

PT5: A lot of autistic people say they hate [the questionnaires] because you’ve got to pick a zero, one or two or three, and they’re like, ‘But I’m in between, I don’t understand’.

Therapists also reported a suspicion that observed progress and scores on anxiety disorder measures might not be in agreement because of conflation of arousal and anxiety disorders in the instruments. This is particularly troublesome when trying to make sense of outcomes for an autistic client:

PT5: Basically [autistic peoples’] anxiety scores remain quite high, so even though they’ll say ‘Yeah, I’m getting out and doing more, feeling more confident’, they’re still scoring off the scale. So, whether that’s an issue of mislabelling arousal levels as anxiety or question of how we’re measuring it, we’re not measuring symptoms accurately.

Therapists also reported that autistic people sometimes found the measures vague, confusingly abstract, or even self-contradictory:

PT4: [S]ometimes an autistic client will read the question . . . [and] say ‘I don’t know how to answer that honestly because what do you mean? Can you give me examples?’ And that’s where I think it can fall down.

The ‘structural’ aspect of the NHS TTad service that participants identified as most obstructive to effective work with autistic people was the time-limited nature of the therapy itself. Autistic people were seen to benefit most when they could answer questions without feeling rushed. Given the structured and time-limited therapies used, NHS TTad therapists saw themselves as rarely able to provide the time needed.

Time was viewed by most participants to be a particular problem when conducting (usually telephone-based) assessments:

PT12: When you do the . . . training there’s a full module on how to assess people in [TTad], and you’re encouraged to do Socratic questioning, but we all know that we don’t really have that much time to do that properly when assessments are half an hour.

While some participants maintained that these problems were magnified with autistic people, others argued that the opposite might be the case:

PT1: It’s not a very long assessment and it just does what it does to look at what the symptoms are at the time and allocate . . . Probably because it’s simple and straightforward, it might be helpful for autistic people.

On the matter of available time for reflection, consolidation, and preparation, however, there was a great deal more unanimity among participants:

PT10: I think there’s not nearly enough time for reflection in [TTad]. We’re probably working with more complex people than we were intended to be working with, and I think they all take time to reflect and prepare for.

### Theme 3: therapeutic environment

A key strength raised by some participants was the ability to offer a therapy via face-to-face interaction, virtual spaces, or telephone:

PT11: When I’ve had [assessments] through for people who have been autistic, [they] will ask for face to face. So, I’ve done some face-to-face assessments because they preferred them to being over the phone.

PT2: . . . working with [an autistic person] on the phone rather than face-to-face . . . was helpful for that particular person because they struggled with the face-to-face interaction.

Telephone assessment was the default approach within NHS TTad and, although the general lack of nonverbal cues when using the telephone was cited as a therapeutic obstacle by some, scheduling and organising face-to-face work was more inherently time-consuming within an already time-pressured system:

PT1: [A client] that I assessed the other week, she ended up having to wait longer to be seen face-to-face . . . because it’s harder to [get] assessment slots into someone’s face-to-face treatment diary . . . everyone’s out of the habit of booking [and] we have so little face-to-face time.

Some participants reported that autistic people particularly benefit from the presence of family members or other sources of support during assessments or therapy:

PT2: . . . if we know that [a client is] autistic to start with, we could think about making reasonable adjustments at that point . . . we might think about . . . a face-to-face assessment . . . or having somebody else present.

Further scheduling issues were created when additional attendees are required at a face-to-face appointment.

The final problem raised by participants around face-to-face work with autistic people related to the physical spaces available for therapy. These were, in some cases, viewed to be particularly unsuitable (even unpleasant) for individuals highly sensitive to stimuli in their immediate environment:

PT10: You work in some terrible places, and . . . I think ‘Oh my God, this is horrible’. You know, people just kind of don’t seem to notice. But I think for autistic people it’s a bigger issue.

However, some participants identified autistic people for whom CBT had worked well. This was chiefly on the grounds of the structured nature of CBT:

PT7: I’m thinking of this particular person who engaged extremely well. I think some of his autistic characteristics actually made him do better with the therapy. He attended every time, he attended bang on. If I asked him to do something, he did it and . . . those are the main things you need to do well with the CBT-based approach, in my view.

A key aspect to the contrary view, meanwhile, was that cognitive restructuring was often much harder for autistic people:

PT1: [T]hat rigidity of thinking is there because it’s a neurodiversity. There’s no point trying to . . . I’ve got to work with that, and the client has got to work with that, and we’ve got to find a way to help them live a life with it rather than trying to, you know, challenge the rigidity of their thinking.

Similarly, it was widely considered that specific life events may not be ‘triggers’ to depression or anxiety disorders in the same way in autistic people as in more familiar populations to the participants:

PT2: It’s not always that something has happened and that’s what the reaction is but . . . it was my assumption [although] I knew this person was autistic, and I knew that was a big part of the presentation, I was still listening out for what’s happened. What’s the explanation? I think that the explanation was linked to the autism rather than any of the trigger in the last week.

Ultimately, however, there was a generally held concern that any approach which tried to ‘restructure’ the way that an autistic client thought about the world might end up exacerbating anxieties:

PT7: I suppose my greatest fear is making the [client] feel worse than [they] did in the first place . . . [C]ognitive restructuring, I’m now realising, is the one I feel on very shaky ground [with] and actually I would hesitate to engage in that intervention with someone with autism.

Other forms of therapy, such as EMDR, were reported by some as effective. This may initially appear surprising given its highly manualised approach. The core differences between CBT and EMDR, in both form and function, however, were systematically unpacked by PT12:

CBT is protocol-driven, but actually . . . you have to be more adaptive, but with the EMDR . . . it’s contained, it’s concrete, whereas perhaps CBT is a little bit less concrete . . . CBT [is] about black and white thinking, and actually looking for the grey . . . and in my experience people [who] are autistic, don’t like grey, they like black or they like white. So, I think that’s kind of why EMDR, for me personally, seems to work quite well.

The use of psychoeducational elements in therapy were also reported to have been very productive in working with autistic people:

P6: . . . just having that general understanding of what sort of areas might be difficult for [autistic people] and then, importantly, taking the emphasis off ‘What’s wrong with me?’ That psychoeducation thing of ‘Well, you know, it’s quite normal. You would find it hard in social situations because you have autism’ and building up the strengths . . . Being compassionate to self and focusing on these are my strengths as someone with autism, this is what I’m really good at which other people aren’t good at? That’s certainly I think a good position to have.

### Theme 4: training and adaptations

As highlighted elsewhere in this analysis, there was a unanimous concern that training around autism and working with autistic people in NHS TTad has been quantitatively and qualitatively lacking:

PT3: [M]y experience was that we didn’t really get the training before we got the [autistic people] through the door. So, it was kind of, this is something I’m dealing with. And I suppose as therapists, we [were just] expected to make adaptations to kind of fit.

Some participants did note that autism training had recently become a higher priority, with demonstrable benefits for not only working with diagnosed autism but also recognising potentially undiagnosed cases:

PT2: I’ve had some training recently . . . well two lots of training about the same time on autism, and then I don’t know if I I’m seeing it more because I know about it, but I do feel like I’ve had at least two, four autistic people on my caseload at a given time over the last year or two. So I think generally speaking, the diagnosis itself is getting wider and perhaps there is more awareness than there was before, but me having done this training and having more awareness meant that I wasn’t just seeing people who . . . had a diagnosis as being autistic, but I was also able to identify people who might be autistic, and then go through the questions to think about whether that was a meaningful label for them.

Among the participants for whom autism training was still seen to have been minimal, it was the ‘unknown unknown’ aspects of autism that most eroded their professional confidence:

P6: I think most, many therapists know, myself included, we don’t have a clue. You know, we don’t have a clue what to do, where to start. It can be quite anxiety provoking . . . [if] autistic people are gonna end up in [TTAD] services, cause there’s no other autism-specific service, then . . . actual training would be good.

The areas in which participants specifically understood that they needed improved knowledge and skills for practice were chiefly around interpersonal communication techniques, adaptation skills and how to separate mental health conditions (not least social anxiety) from autism itself:

PT3: I feel the training that would be more suitable would be about how to deliver CBT or IPT to somebody who either believes they’re autistic or has that diagnosis and how that differs.

PT10: . . . having a bit more of a framework to think about in terms of . . . timings, ways of working, what help, what might not be so helpful. Ways of wording things. Because I think I probably use quite a lot of metaphor, which is probably confusing [for autistic people], isn’t it? So yes . . . I would love to do some more training on [this].

Finally, stakeholder involvement in training was discussed; and it was identified that training was not always co-produced and/or delivered with autistic people:

PT3: I suppose the only thing that I would think that would be helpful would be and I don’t know how likely this would be to happen, but actually to hear from that client group themselves. So often the training is delivered by, as far as I’m aware, people who don’t have autism . . .

While the quality and quantity of direct training within NHS TTad was viewed largely negatively, most participants did impress that they had made adaptations successfully with autistic people, largely through aggregated experience over time and independent research, often augmented by trial-and-error:

PT6: I’m certainly a lot more confident than I was and I think that probably, certainly within [TTAD], I’ve probably now enough of a sense of what’s helpful and how to do things, and also I have got access to some resources both from CPD and from my own interest in autism . . . after having worked since 2016. So that’s what, seven years?

The first routinely described adaptation related to maintaining a focus on autistic people’s concrete concerns and breaking questions down into smaller components. For example,

PT12: [I]t was about, you know, breaking that [question] down. So, what is it about cleaning the teeth that is really difficult? . . . Is it the smell? Is it the taste? Is it the touch? Is it the, you know, the sensation having the toothbrush in? Is it the sensation of the toothbrush? Is it other idea having something in your mouth?

Second therapists also discussed the normalisation and/or validation of autism itself during therapy:

PT6: That’s very useful, so normalising you know, you would feel like that if you have autism, that’s what people with autism experience, that’s normal. I think that’s a very important part of it.

Therapists also reported carefully monitoring how the autistic person interacted with the physical environment, and then making constructive changes:

PT2: [A client] that I worked with and had a sensitivity to the light being on, the bright sort of regular office light that you have on and we had the lamp, so we turned off the big light to put on the lamp and that was a reasonable adjustment to support the needs that they had . . . so, you know, there might be some relatively simple things that we can do that’s gonna meet the needs of that person . . . make adaptations just to make sure that they can engage in the process, really.

There was a broad consensus that NHS TTad supervisors had been a strong and supportive resource, in some cases being trusted to find additional resource in the system where it would most benefit a client:

PT3: I don’t know if this is [NHS TTad] or if this is my service [but] if I went to my supervisor and said, ‘You know, I’ve identified that I’m working with somebody who either identifies as potentially having autism or has the diagnosis, and what we’ve agreed is that actually more time or an extra session or more frequently or a longer session’, I’m pretty confident that . . . they would say ‘Yeah, absolutely. Let’s, let’s meet that need’.

Finally, the value of group supervision was raised as a potential augmentation to current practice, in terms of helping learn about how to support autistic people:

PT8: [T]hings like group supervision might be good, you know, because obviously we have individual supervision, [but] group supervision, where you can bring a case and you can discuss it and people can understand together, you know . . . what you did right, what you did until what happened, you improve it and exposure to that would probably be really good for therapists and for us all to learn more.

## Discussion

This study has presented a qualitative analysis of the experiences of psychological therapists when working with autistic people within English NHS Talking Therapies services. Twelve therapists were interviewed, and four global themes were identified: (a) Experience and Trepidation, (b) Wrong Service, Only Service, (c) Therapeutic Environment and (d) Training and Adaptations. Participants routinely addressed the importance of identifying autism in therapy, the challenges of managing the expectations of autistic people about therapy, the challenges and benefits of the service structure, the impacts of a time-limited structure on autistic people, and the therapist’s own need for focussed training and supervision support. There are similarities in the issues identified in the current study and those identified by studies such as that by Riches et al. (2023), such as the challenges of expectation management and pacing, understanding how emotions and social relationships operate in the lives of autistic people, and the challenges that rigid service structures can present. All previous studies reporting the experiences of therapists in working with autistic people emphasise the value of therapy-specific autism training and supervision (e.g. Lipinski et al., 2022; Spain et al., 2023).

Research has reported inconsistent outcomes for autistic adults around conventional talking therapies, not least CBT (Binnie & Blainey, 2013; Menezes et al., 2022). The data presented above indicate issues in therapy for autistic people that might be important to consider in training design. For example, the issue of a perceived ‘rigidity’ of thinking among autistic people was variably identified by participants as both an obstacle to progress and a facilitator of it, depending upon how it was (or was not) therapeutically harnessed. This observation underscores how future training for therapists addressed needs to avoid a ‘one size fits all’ model of therapeutic direction with autistic adults.

Training and supervision for therapists who work with autistic people, and its affirmative impact upon knowledge about autism and attitudes towards autistic children and adults, has been well documented in recent research (Alaghband-rad et al., 2023; Corden et al., 2022) and across a range of contexts such as general medicine (Clarke & Fung, 2022), school teaching (Alexander et al., 2015), high security mental health inpatient services (Murphy & Broyd, 2020) and policing (Railey et al., 2020). In England, as noted previously, training for work with autistic people and/or people with an intellectual disability is now considered mandatory across public Health and Social Care sectors. An initial evaluation of this training initiative (National Development Team for Inclusion, 2022) indicates that it was well received, led to an increase in knowledge, skills and confidence in working and communicating with autistic people, and was used by most to make active changes in practice. This research makes clear, however, that genericism in training around autism has prospective limits on its usefulness; for participants in the current study, training around specifics, particularly in how autism might affect given aspects of practice, was typically regarded as most useful.

The analysis above further indicates that participants were typically experienced, and routinely willing (if not always fully equipped) to adapt their therapeutic technique for autistic people. This is consistent with research on NHS TTad therapists’ adaptations for other client groups (e.g. Dagnan et al., 2023) and such flexibility is considered a core CBT competency (Roth & Pilling, 2008). However, the challenges reported by participants in this study suggest that focussed and technical training on autism-specific interventions is necessary if the gap in therapeutic outcomes for autistic and non-autistic people in NHS TTad is to be closed. Participants in the current study also noted the centrality of supervisors (Maddox et al., 2020) in NHS TTad, and a focus on targeted supervision and the skills of supervisors will be important.

El Baou et al. (2023) highlight that TTad outcomes for both autistic and non-autistic populations are positive, but more positive for the latter (a 56.1% rate of reliable improvement compared to 61.7%). Moreover, they report that the factors associated with positive outcomes for people who are not autistic (such as being employed and in higher socio-economic groups) did not predict outcomes for the autistic group. The finding of different predictors adds weight to the suggestion that therapies and structures for this therapy delivery model may need adaptation for autistic people. However, the relatively small gap in outcome reported by El Baou et al. (2023) may also imply that these services are more successful for autistic people than therapists realise.

### Study limitations

While sample size and general representativeness are not typically key concerns in qualitative research (Malterud et al., 2016), this study was nevertheless grounded in a relatively large and detailed qualitative body of data drawn from therapists working (as aforementioned) in both urban and rural TTads, and within a relatively standardised service. Moreover, although geographically small on the global scale, England is noted for its cultural and administrative diversity, and the north is broadly subject to higher deprivation and lower healthcare funding per capita than the south (National Institute for Health and Care Research [NIHR], 2024). Consequently, findings should be contextualised in these terms.

## Conclusion

Autistic people are more likely to experience depression and anxiety disorders than the general population, and their experience and presentation of these disorders is likely to be different from those of the general population. Therapists in NHS TTad did not always feel well equipped to separate aspects of autism itself from that which emerges as a mental health disorder in an autistic person. This specific issue occasionally left the participants in this study feeling powerless, or as if they had done more harm than good. It is the case, however, that statistical outcomes of TTad services in England indicate a broadly positive therapeutic environment for autistic people with depression and anxiety disorders. In these terms, and in any initiative moving forward, a research-led perspective on what therapists are doing right, such as that articulated by O’Brien and colleagues (2024) regarding psychologist–client relationships in Australia, is important to counterbalance that which they might feel they are doing wrong. Where the gravity of success is broadly positive, emphasis on a deficit model is seldom productive.

## Supplemental Material

sj-docx-1-aut-10.1177_13623613251341610 – Supplemental material for ‘Sometimes I’m feeling baffled and they’re probably feeling baffled’: On the experiences of psychological therapists working with autistic people in a structured primary care service for anxiety disorders and depression Supplemental material, sj-docx-1-aut-10.1177_13623613251341610 for ‘Sometimes I’m feeling baffled and they’re probably feeling baffled’: On the experiences of psychological therapists working with autistic people in a structured primary care service for anxiety disorders and depression by Paul K Miller, Samantha LJ Bowden, Natalie Dewison, Barry Ingham, Richard Thwaites and Dave Dagnan in Autism
