# Supplementary material for: ‘Sometimes I’m feeling baffled and they’re probably feeling baffled’: On the experiences of psychological therapists working with autistic people in a structured primary care service for anxiety disorders and depression
Source: Autism. 2025 May 22;29(10):2477–88. doi: 10.1177/13623613251341610 (PMC12417598; doi:10.1177/13623613251341610)
Supplement: sj-docx-1-aut-10.1177_13623613251341610 – Supplemental material for ‘Sometimes I’m feeling baffled and they’re probably feeling baffled’: On the experiences of psychological therapists working with autistic people in a structured primary care service for anxiety disorders and depression [file sj-docx-1-aut-10.1177_13623613251341610.docx]

**Autism & IAPT Services: Therapist Interviews**

**Draft Schedule**

- Scene-setting: Could you tell me a little about the type of work you do and your general experience of adapting therapy to meet the needs of the clients you are working with?
- Opening: Can you please describe your broad experience of working with autistic people?
  - How often, for how long, and to what ends?
  - Can you remember when you were first aware that you were (or would be) working with autistic people and what your expectation of working with this group was?
- Context setting: Using answer from the question above, explore how participant identifies autism and autism-like needs.
- Context setting: Have you experienced any general differences in the life experiences of autistic people that affect the way they specifically present with depression and anxiety? Please give concrete examples from practice if you can.

***Main Interview***

- When it comes to working with autistic persons in IAPT services, what are the core skills/approaches that are generalisable/applicable from your current skillset?
- Where do you feel skills could be developed to better support autistic individuals?
- Does need to develop any particular skills reflect a different approach required when working with autistic individuals? If so, why is that
- When you’ve had good outcomes from this work, what factors in your core therapeutic approach have influenced this, and why? [Examples]
- When you’ve had worse outcomes from this work, what factors in your core therapeutic approach have influenced this, and why? [Examples]
- What are the key challenges you face in working with this group?
- When you’ve had good outcomes with this group, what has influenced a positive alliance and engagement, and why?
- When you’ve had worse outcomes with this group, what has influenced a negative alliance and engagement, and why?
- What are the structural features of IAPT that support you working with autistic people, e.g. the procedures, processes and guidance you follow, and why?
- What are the structural features of IAPT that can impede you working with autistic people, and why?
- When you’ve had good experiences, what aspects of IAPT have particularly helped? [Be specific]
- When you’ve had less productive experiences, what aspects of IAPT have been unhelpful or even obstructive? [Be specific]
  - What features of IAPT do you think need to be adapted or reformed in the light of this?
- What aspects of the assessment process have been most helpful when working with autistic people, and why?
  - Is this dissimilar to when working with neurotypical people? If so, why?
- What aspects of the assessment process have been most difficult to apply when working with autistic people, and why?
  - Is this dissimilar to when working with neurotypical people? If so, why?

***Wrap-Up***

- Summarise key findings for participant.
- Ask for any corrections or further details they wish to add.
- Thank them, provide Debrief sheet, remind of post-hoc withdrawal mechanism and means of access to findings.
